# Saccades to Explicit and Virtual Features in the Poggendorff Figure Show Perceptual Biases

**DOI:** 10.1177/2041669517699221

**Published:** 2017-04-21

**Authors:** Barbara Dillenburger, Michael Morgan

**Affiliations:** Max-Planck Institute for Metabolism Research, Cologne, Germany; Division of Optometry and Visual Science, Institute of Health Sciences, City University London, London, UK

**Keywords:** eye movements, imagery, objects and features, perception, perception or action

## Abstract

Human participants made saccadic eye movements to various features in a modified vertical Poggendorff figure, to measure errors in the location of key geometrical features. In one task, subjects (*n* = 8) made saccades to the vertex of the oblique T-intersection between a diagonal pointer and a vertical line. Results showed both a small tendency to shift the saccade toward the interior of the angle, and a larger bias in the direction of a shorter saccade path to the landing line. In a different kind of task (visual extrapolation), the same subjects fixated the tip of a 45° pointer and made a saccade to the implicit point of intersection between pointer and a distant vertical line. Results showed large errors in the saccade landing positions and the saccade polar angle, in the direction predicted from the perceptual Poggendorff bias. Further experiments manipulated the position of the fixation point relative to the implicit target, such that the Poggendorff bias would be in the opposite direction from a bias toward taking the shortest path to the landing line. The bias was still significant. We conclude that the Poggendorff bias in eye movements is in part due to the mislocation of visible target features but also to biases in planning a saccade to a virtual target across a gap. The latter kind of error comprises both a tendency to take the shortest path to the landing line, and a perceptual error that overestimates the vector component orthogonal to the gap.

The term illusion, because of its connotations of magic and deception, neither of which is germane to the topic at hand, shall be restricted to the title. (Weintraub & Schneck, 1986, p. 147)

## Introduction

**Figure 1.**
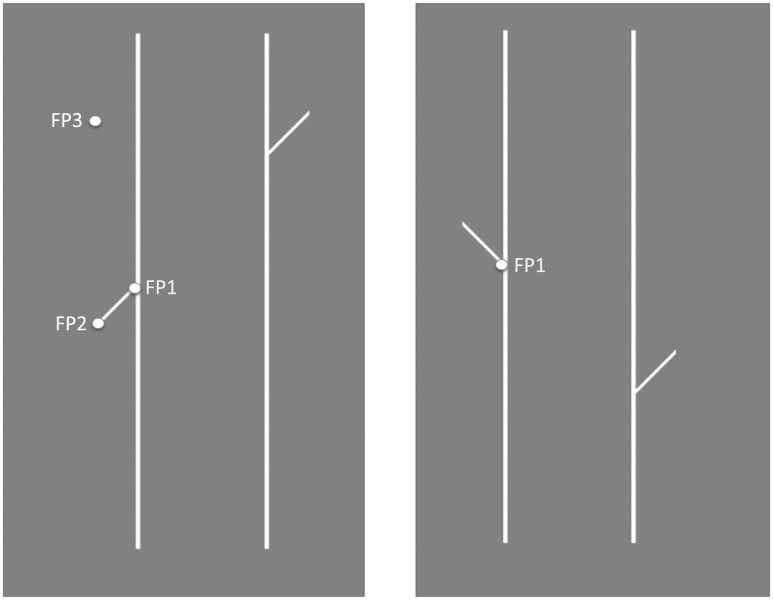
Examples of stimuli used in the experiments. The fixation point (circle) was presented first, followed by the rest of the figure after 1,500 ms. In the left-hand figure, the two pointers were collinear; in the right-hand figure, they were orthogonal. The fixation points used in Experiment 1 are labeled “FP1”; in Experiment 2 as “FP2”; and in Experiment 3 as “FP3.” In all cases, the task was to make a saccade from the fixation point to the right-hand vertical (the “landing line”).

In a recent article (Morgan & Dillenburger, 2016), we described psychophysical investigations of the famous Poggendorff effect. The effect is illustrated in the left-hand configuration of Figure 1. Most readers will see the right-hand oblique *pointer* displaced upwards from the point of perceived collinearity with the left-hand pointer. Similarly, in the right-hand configuration, the left-hand pointer seems to point not at the right-hand intersection of the 45° oblique and vertical but toward the middle of the 45° oblique.

As in the earlier article (Morgan & Dillenburger, 2016), we avoid the loaded term *illusion* in favor of *bias* and we refer to the *P-bias* as any bias in the perception of collinearity in the same direction as that seen in the traditional, upright 4-line Poggendorff figure. The P-bias could arise from many causes, including changes in the perceived angle of the pointers (Blakemore, Carpenter, & Georgeson, 1970) and changes in the perceived location of the acute angles in the figure (Morgan, 1999). Biases in location of the angles could result from neural blurring in first (Glass, 1970) or second-order filters (Morgan, 1999) that place the centroid of the blurred intersection inside the acute angle. Evidence for a location shift was found (Morgan, 1999) using the rather difficult task of matching the perceived orientation of the virtual line between the two intersections to that of a grating. Morgan and Dillenburger (2016) also found evidence in a rigorous two-alternative forced choice task for a location shift, using a 2-dot probe stimulus, but the effect was small and not present in all participants.

In this article, we attempt to provide further evidence for mislocation by measuring the endpoint of saccades made to an oblique T-intersection (Experiment 1). This approach has already been used with success to investigate other positional biases. A well-established perceptual bias (Obonai, 1931) is that the pointer actually aligned with the tip of the 45° target line seems to point toward the *center of gravity* of the target line. Findlay and Hotopf (1985) found that this was also true for a saccade directed toward the tip of the target line.

To measure the putative mislocation bias, we compared the collinear and orthogonal versions in Figure 1 (Experiment 1). The mislocation bias predicts opposite shifts in the two cases. To determine whether the mislocation bias was sufficient to account for the P-bias in saccades, we measured the latter with the right-hand pointer absent and a virtual target on the right-hand vertical (Experiment 2; see Melmoth, Grant, Solomon, & Morgan, 2015). Finally, to determine to what extent the saccade bias was due to a tendency to take the shortest path to the landing line, we varied the saccade starting point (Experiment 3).

## Materials and Methods

### Apparatus

The experiments were carried out in parallel on different subjects (see later) in two different locations, one in City University and the other in the Max-Planck Institute for Metabolism Research, Cologne. In both cases, eye recording was carried out using the same model of the EYELINK 1000 system (see later). Also in both cases, stimuli were generated by identical scripts in Matlab on the same model of MacBookPro computer, running MATLAB and using the PSYCHTOOLBOX PTB3 software (Brainard, 1997). In Cologne, stimuli were presented on the screen of SONY Trinitron monitor with resolution 1400 × 1050 pixels and viewed at 75 cm so that 45 pixels subtended 1° of visual angle. The background screen luminance was 16 cd/m^2^, with average luminance of the stimulus components being 49 cd/m^2^. In London, stimuli were presented on a vertically oriented Protouch 17-in. TFT flat-screen display. On-screen pixel size was 0.36 mm, and average background luminance was 55 cd/m^2^, while average luminance of the stimulus components was 130 cd/m^2^. The difference in geometry between the two displays was taken account of in the eye movement analysis.

### Stimuli

Both collinear (left hand; Figure 1) and orthogonal (right hand; Figure 1) pointers were used, in different blocks. In addition, in half the trials within a block the up-down orientation of the figure was inverted. The vertical position of the pointers was jittered over trials from a uniform distribution of displacement between 0 and sqrt(2) × P, where P was the pointer length, in order to prevent stereotyped responding, such as choosing the middle of the landing line as the target. On a given trial, the two pointers were displaced by the same amount, so they were always collinear or orthogonal.

The pointer orientation (as shown in Figure 1) was 45° or 135° when the figure was inverted. The length of the verticals was 600 pixels (12.5° VA), and their separation one quarter of this (3.13° VA). Pointer length was 70 pixels (1.47° VA). The line thickness for both pointer and inducers (verticals) was 5 pixels (6.25 arcmin).

### Subjects

The subjects in London were AJ a male PhD student and JS an experienced psychophysical observer. In Cologne, the subjects were the first author (BD), a naïve paid subject (DW), a psychophysically experienced postdoctoral fellow (KS), and an experienced PhD student (NN). Two subjects carried out some conditions in Cologne and some in London: MM (authors) and JF a PhD student. Subjects KS, NN and JF were also naïve as to the purpose of the study. Informed consent was obtained prior to inclusion, and procedures were in accordance with the Declaration of Helsinki.

### Procedure

In all the experiments, subjects were seated comfortably in front of the monitor with the head loosely restrained by an EYELINK head and chin rest, and with two fingers resting on the response key of a keyboard. Each session began with an EYELINK calibration (see later) followed by 60 trials in a single block. Each trial began with the presentation of a fixation point (FP) for 1500 ms. Then the stimulus appropriate to the experimental condition was presented on the display screen, and the subject made a saccade according to instructions that had been given and illustrated before the experiment. The subject’s task was to make a saccadic eye movement as quickly as possible to the Target. In Experiment 1, the target was the opposite (Figure 1) intersection of vertical line and pointer. The instructions were to “make the saccade as quickly and as accurately as possible.” Once the saccade had been recorded (see later) or alternatively if no saccade was recorded for 1 seconds, the stimulus was replaced by a blank gray screen. The subject pressed a response key to initiate the next trial. In Experiments 2 and 3, the target was the extrapolated point of intersection of the left-hand pointer and the right-hand parallel (the landing line).

### Eye Movement Recording

Eye movements were recorded at 1000 Hz with Eyelink 1000 (SR Research) using chin and forehead rest to stabilize the subject. To record primarily saccades relevant to our question and thus to speed up our exclusion procedure, we set Eyelink parameters for velocity threshold (40°/s), acceleration threshold (8,000) and motion threshold (0.5) to focus saccade detection on larger saccades and specifically exclude microsaccades. For example, a velocity threshold of 22°/s allows detection of saccades of 0.3° amplitude (Zimmermann, Burr, & Morrone, 2011), whereas a larger threshold (e.g., 40°/s) reduces the number of microsaccades detected (cf. Collins, Semroud, Orriols, & Doré-Mazars, 2008).

Each experiment was started with a 9-point calibration (Eyelink), that is, nine targets were presented successively at locations covering the entire screen. When fixations to each target appeared stable, they were accepted manually by the experimenter. Good quality calibration was ensured by a standard validation (Eyelink), in which nine targets, slightly offset to the calibration target positions, are presented, and fixation to each is again accepted manually. Based on the Eyelink validation process’ feedback (overall distance of validation fixations to the expected gaze positions), the calibration was accepted or repeated.

**Figure A1.**
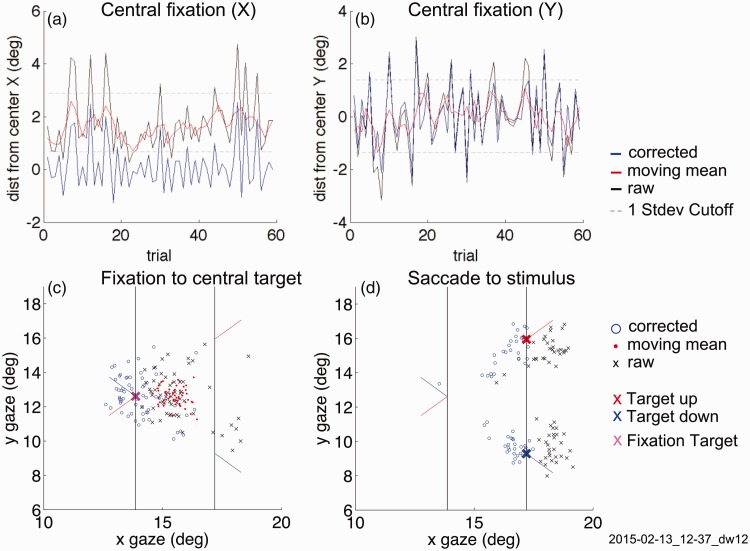
Example of drift and head movement correction of a single test run with 60 trials. (a) Horizontal distance (gaze coordinates, degree of visual angle) of subject’s fixation across trials from the calibrated central fixation target. (b) Vertical distance (degree) of subject’s fixation across trials from the calibrated central fixation target; black traces in (a) and (b) show the raw eye movement data, red traces show the moving mean (smoothing window of five trials, cropped to one standard deviation of the mean, as indicated by the horizontal dashed lines) over all 60 trials. The blue trace shows the corrected gaze positions. In (c) and (d), initial fixation (c) and endpoint of the first saccade (d) are shown superimposed on the stimulus configuration (Experiment 1), with gaze coordinates plotted in degree. Raw eye positions are shown as black crosses, moving mean thereof in red (c), and corrected eye positions as blue open circles (c) and (d). Initial fixation location is indicated by the pink cross (c), saccade targets as red (upward condition) and blue (downward condition) crosses (d).

Ideally, the recorded eye position when the subject is fixating in the center of the screen at the start of each trial should agree with the center of the screen measured during the calibration procedure. However, this is not always the case: For example, a comparison of systematic errors in eye tracking across 16 subjects has shown systematic offsets across different target locations within the range of +/− 2° of visual angle (Hornof & Halverson, 2002). In a comparable condition (Experiment 2, saccade from a fixation spot to a single target point), our eight subjects showed comparable offsets during initial fixation from calibration of on average 1.44° + 0.83 (X) and −0.15° +/− 0.58 (Y). To correct for these offsets and any slow drifts occurring during the recording, we conducted a correction step described in detail in Appendix Figure A1.

Velocity profiles (calculated at sampling frequency, i.e., 1000 Hz) were filtered using a second-order Butterworth filter with cutoff frequency of 100 Hz (cf. Becker & Juergens, 1990; Van Opstal & Van Gisbergen, 1987).

**Figure A2.**
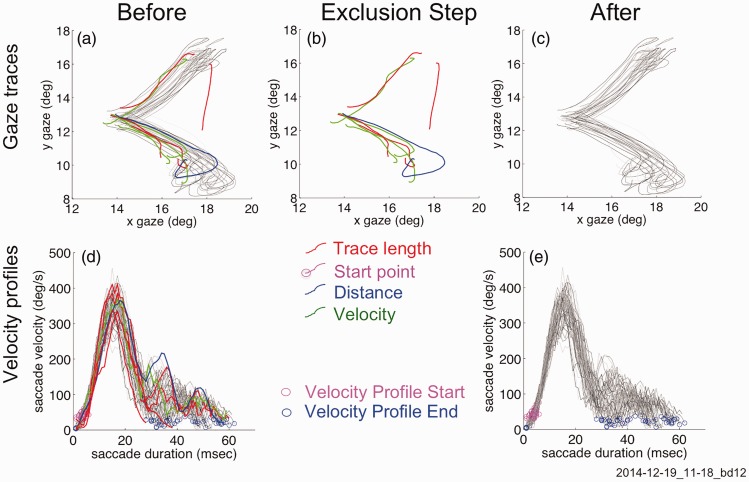
Exclusion procedure. Saccade trajectories of a single dataset (subject B. D., Experiment 1, 60 trials) are plotted in gray (a) before and (c) after the exclusion step in the top row. Velocity profiles are shown in gray, (d) before and (e) after exclusion, respectively, in the bottom row. In the middle graph, (b) trajectories of excluded saccade are plotted colored according to the parameter in which they deviated by more than two standard deviation from the average saccade: red: trace length; pink: starting point; blue: summed distance; green: peak velocity or summed velocity. Note that traces are color-coded according to the first deviating parameter, if they differed from the average in several aspects. In (d) and (e), velocity profile-based saccade starts are marked as pink, saccade ends as blue open circles.

Before any further analysis, we conducted an exclusion step to select only eye movements that had occurred after a minimum latency (saccade start at initial target onset + 80 ms), and to exclude blinks and outliers from the saccade data. Trials were also excluded if saccades deviated from the overall saccade population in amplitude, start position, or velocity profile parameters. Details of the exclusion procedure are described in Appendix Figure A2.

To analyze saccade data across conditions and subjects, we calculated the average saccade endpoints. Endpoints of individual saccades were defined as the point at which the saccade’s velocity dropped below 30°/s, plus 4 ms (following Van Opstal & Van Gisbergen, 1987). In saccades in which velocity did not reach the 30°/s threshold following velocity peak but continued in, for example, a multiple peak profile (i.e., short latency correction saccade), or maintained a steady velocity higher than 30°/s (i.e., turnaround saccade), we estimated the primary saccade’s end. To do so, we calculated the slope of the deceleration of the velocity profile between peak velocity + 4 ms (to catch only the steep slope) and the timepoint at which velocity had dropped to 50% of the peak velocity. This slope was used to extrapolate to the 30°/s threshold.

As the median is less affected by outliers (as common even in corrected eye movement data) and represents the center of skewed distributions better than the mean, we used the median of all individual saccade endpoints for analysis in this study.

## Results

### Experiment 1: Bias in the Location of the Acute-Angle Intersection

In the first experiment, subjects were instructed to make saccades as quickly as possible from the initial FP, to the opposite intersection between a vertical line and a 45° oblique. The physical arrangement of the stimuli is shown in Figure 1. In one condition, the obliques were parallel and collinear; in the other, they were orthogonal. The two conditions were run in separate blocks. On half the trials, the configuration was *upright* as in Figure 1; in the other half, it was inverted. The purpose was to see if saccades would fall within the angle, rather than on the actual intersection, as predicted from psychophysical data (Morgan, 1999).

#### Experiment 1 results

**Figure 2.**
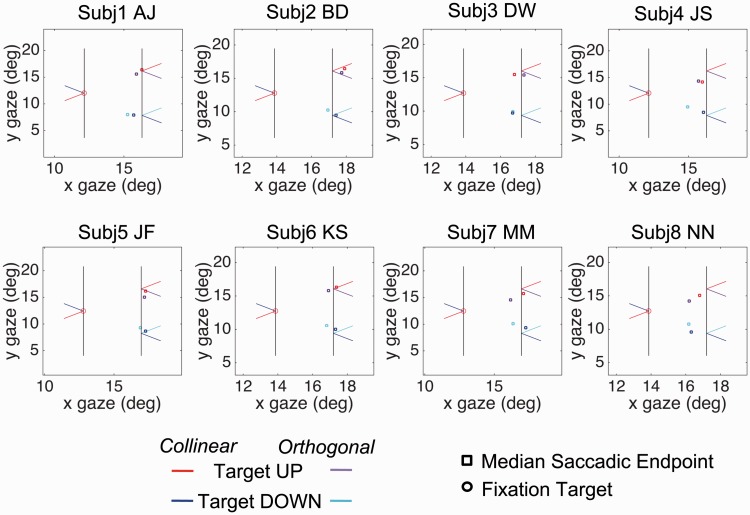
The figure plots the median endpoints of saccades in Experiment 1 as rectangles (red or purple, upwards pointing configuration; blue or cyan, downwards pointing). Initial fixation position is shown as an open circle. Saccades were to target the intersections of right-hand inducing line and pointers. Each panel represents a single subject. The data in the collinear condition are plotted in red or blue, those in the orthogonal condition in purple or cyan.

**Figure A3.**
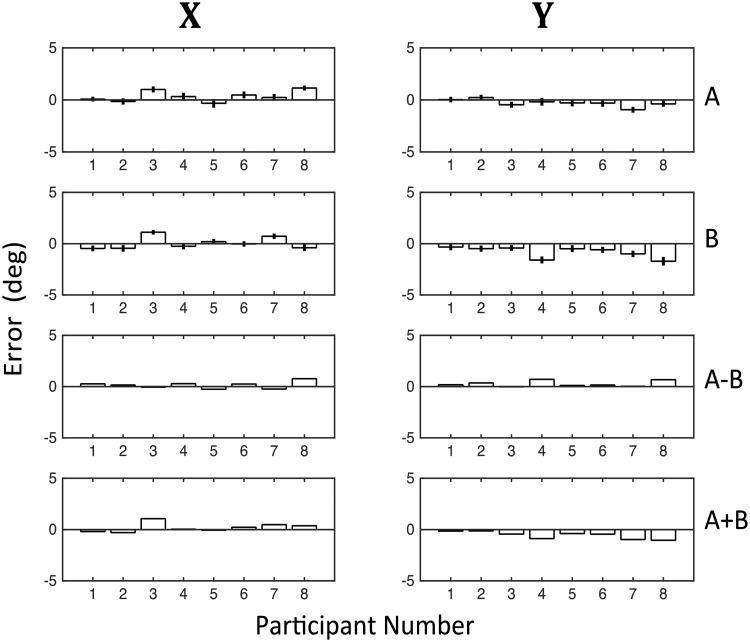
Results of Experiment 1. The figure shows the median and interquartile range of saccadic errors, separately for the X (left) and Y (right) dimensions in space. The results for upright and inverted configurations are combined into a single grand median. Within each panel, the height of each bar represents the error from the nominal target in deg VA for each of the eight subjects who completed all conditions. By convention, negative values mean that the value is less than it would have been had the saccade landed on target. The error bars show interquartile range. The first row shows (a) the results for the collinear configuration (see Figure 4). The second row (b) shows results for the orthogonal configuration. The third row shows the difference Collinear−Orthogonal, a measure of the acute-angle bias without the influence of undershoot (*t* = 2.5, *p* < .05). The fourth row shows the sum Collinear + Orthogonal, a measure of the undershoot (*t* = 3.6, *p* < .01).

Figure 2 shows plots of the median saccade endpoints for individual subjects in an X-Y space with the actual positions of FP, and the target positions also included. The raw data suggest that there is a systematic Y error in both configurations, but that this is stronger with the orthogonal condition than the collinear. (For statistical analysis, see Appendix Figure A3). The error is in the direction of the saccade taking a shorter path across the gap to reach the landing line than it should to meet the target. The fact that this effect is significantly smaller in the orthogonal than the collinear configuration implies that there is also an effect for the saccade to be attracted into the acute angle.

**Figure 3.**
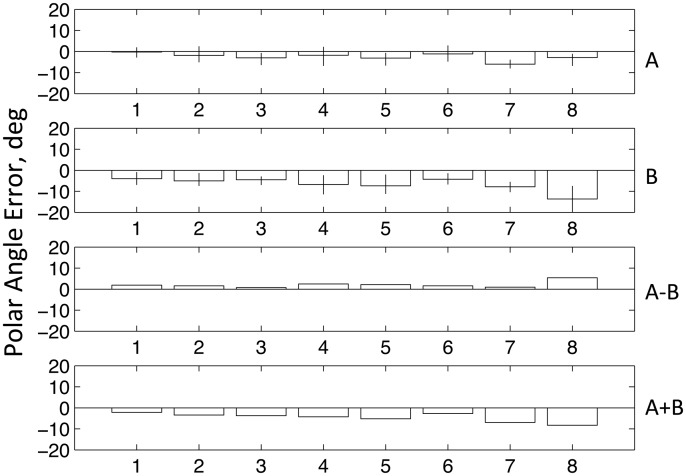
Results of Experiment 1. The figure shows errors in the saccade polar angle with respect to the target angle from fixation (i.e., abs(target) − abs(saccade)). Negative values indicate that the saccade was following a shorter path than the correct trajectory to meet the target. The first row shows (A) the results for the collinear configuration (see Figure 4). The second row (B) shows results for the orthogonal configuration. The third row shows the mean difference collinear-orthogonal, a measure of the acute-angle bias. The fourth row shows the sum collinear + orthogonal, a measure of the general tendency to follow the shortest or horizontal path to the landing line.

Figure 3 shows the difference between saccade polar angle from fixation and target polar angle from fixation, the latter being +/−45°. Saccade angle is calculated as the polar angle between saccade start point and endpoint. We define a negative polar angle as an angle that will produce a shorter path to the landing line. The mean magnitude of this error, (A + B)/2, over the two conditions was −4.52° (*t* = 6.32, *p* < .001). However, it is clear from Figure 3 that the absolute magnitude of this error was greater in the orthogonal condition (B) than in the collinear condition (A). To measure the size of this difference and to test its statistical significance, we take the mean difference (A − B)/2. This revealed a difference of 1.73° (*t* = 4.49, *p* < .001) consistent with a shift in the direction of the saccade into the acute angle. The absolute magnitudes of the errors in the two conditions were similarly significantly different (*t* = 4.49, *p* < .001), which would not have been the case if the errors were equal and opposite. The mean (A + B)/2 plotted Row 4 is a measure of any bias toward the saccade taking a horizontal path. These results confirm the direction of the bias reported from psychophysics by Morgan (1999) and by Morgan and Dillenburger (2016).

### Experiment 2: Biases in Extrapolation

The purpose of the second Experiment was to measure the P-bias with the traditional Poggendorff figure, to compare it with the previous experiment. The physical stimulus was as shown in Figure 1 (left-hand panel) with the FP (“2”) marked except that the right-hand oblique was absent. Participants fixated on this point, with the rest of the figure absent, and once fixation had been verified, the rest of the figure appeared and participants made as rapid a saccade as possible to the imaginary intersection of the left-hand pointer and the right-hand vertical *landing* line. In the control experiment, the left-hand vertical line (the inducer) was absent. This controls for any tendency of saccades to take a horizontal path or a path toward the center of the landing line. A larger bias with the left-hand vertical present would be consistent with a component of the bias due to angular repulsion. However, it would also be consistent with a bias toward taking an orthogonal path between two verticals, rather than across any gap. The difference between experimental and control biases is used to measure the P-bias; the sum of the two biases measures any general tendency to take the shortest path to the landing line.

#### Experiment 2 results

**Figure 4.**
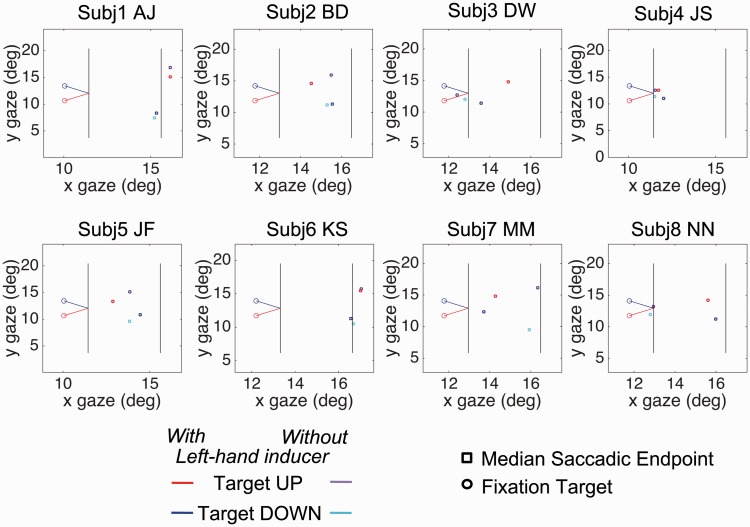
Experiment 2: The figure plots the median endpoints of saccades as rectangles (red or purple, upwards pointing configuration; blue or cyan, downwards pointing, red or blue with left-hand inducer, purple or cyan without). Initial fixation position is shown as an open circle. Target position is the extrapolated landing point of the pointer on the right-hand inducer. Each panel represents a single subject.

**Figure 5.**
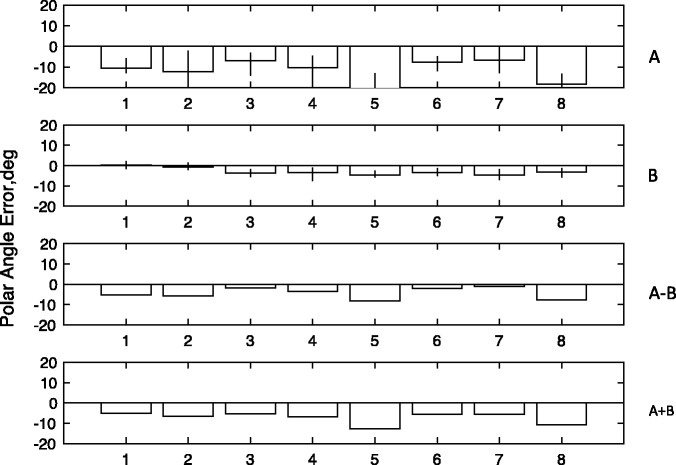
Experiment 2: The figure shows the absolute angular error of the saccade with respect to the target angle from fixation (i.e., abs(target) − abs(saccade)). The top row (A) shows results from Experiment 2, where both vertical lines and the left-hand pointer were present. The second row (B) shows results from the pointer-only control, where the left-hand inducer was absent. The bottom row shows the difference between experimental and control conditions. Otherwise, conventions are as in Figure 3.

Figure 4 shows illustrative graphic plots of the mean saccade endpoints for individual subjects in an X-Y space with the actual positions of FP and the target positions also included. Figure 5 shows the difference between saccade polar angle from fixation and target position from fixation, the latter being +/−45°. Saccade angle is calculated as the polar angle between saccade start point and endpoint.

**Figure A4.**
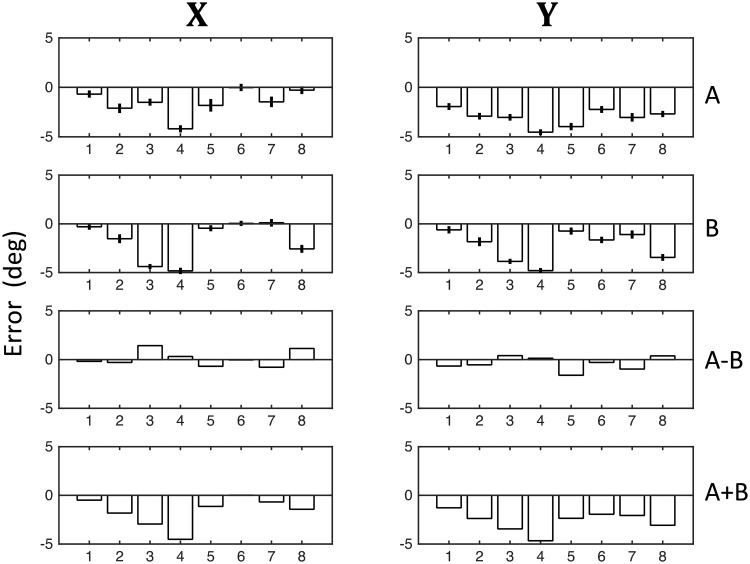
Results of Experiment 2. The figure shows the median and interquartile range of saccadic overshoots, separately for the X and Y dimensions in space. The means for the upward- and downward-pointing configurations are combined into a single grand mean. Within each panel, the height of each bar represents the extent of overshoot from the nominal target in deg VA for each of the six subjects who completed all conditions. The error bars show interquartile range. The first row shows the results for the condition where the left-hand vertical inducer was present. The second row shows results for the control condition where the left-hand vertical was absent. The third row shows the mean difference between the two conditions, a measure of the putative P-bias due to the vertical. The fourth row shows the sum of the two conditions, a measure of undershoot. The figure shows that there were undershoots in the expected direction both with the left-hand vertical inducer present and absent. However, these are largely accounted for by a general undershoot (A + B)/2 rather than by a P-bias (A − B)/2. The X shift due to the inducing line (A − B) was 0.114° VA (*t* = 0.5, *p* = .7), the Y shift was −0.39° VA (*t* = 0.2, *p* > .5). The overall undershoot (A + B) was −1.6 (*t* = 2.05, *p* < .5) deg VA for X and −2.65 (*t* = 3.29, *p* < .001) deg VA for Y.

Figure 4 shows that there were massive Y errors for most subjects, in the direction expected from the P-bias. (Use a ruler to verify this rather than relying on biased perceptual extrapolation!). There were also errors for some subjects in X, which were unexpected because the subjects were instructed to move their eyes to the landing line. (For statistical analysis, see Appendix Figure A4). The error was in the direction of stopping short of the landing line. In some cases, this X error was dramatic. Subject JS in particular hardly moved his eyes from the FP at all, although he thought he had.

Analysis of saccade polar angle revealed (Figure 5) large biases in the saccade polar angle, in the direction of the saccade taking a shorter path to the landing line. This general bias (A + B)/2 was −8.9° (*t* = 12.57, *p* < .001). In addition, however, there was a significant P-bias of −3.77° (*t* = 2.89, *p* < .05) caused by the inducer line (A + B)/2.

Because of the anomalously short saccades made by J. S., the analysis of polar angles was repeated with this subject eliminated. The difference A − B was still significant, *t*(6) = 4.16; *p* < .01, as was the mean A + B, *t*(6) = 6.36; *p* < .01, and the absolute difference abs(a) − abs(b), *t*(6) = 4.2; *p* < .01.

### Experiment 3

Experiments 1 and 2 have shown an overall tendency of saccades to follow a shorter path to the landing line than the path to the target. This would be consistent with a “least effort; principle.” However, an alternative interpretation is that saccades made across a gap between two lines are determined by a *perceptual* path that is shortened. In other words, a perceptual process constructs a virtual target on the landing line that becomes the target of the saccade, and this construction is subject to a P-bias. These two possible interpretations can be separated by varying the position of fixation before the saccade (Figure 1). The FP3 was placed at 45° from the target, like FP2, but in the opposite direction. (Using trigonometrical convention, FP3 is 135° and FP2 is at 315°). The eye movement begins at FP3 instead of at FP2, a perceptual effect will lead to a *longer* saccade. To test this, the next experiment compares the FP2 and FP3 conditions.

The configuration for Experiment 3 is shown in Figure 1 (earlier) with the FP3 superimposed. As in Experiment 2, the subject’s task was to move as quickly and accurately as possible from the FP to the imaginary intersection point of the left-hand pointer on the right-hand vertical; but now the expected P-bias would lead to the saccade taking a longer path to the landing line.

#### Results

**Figure 6.**
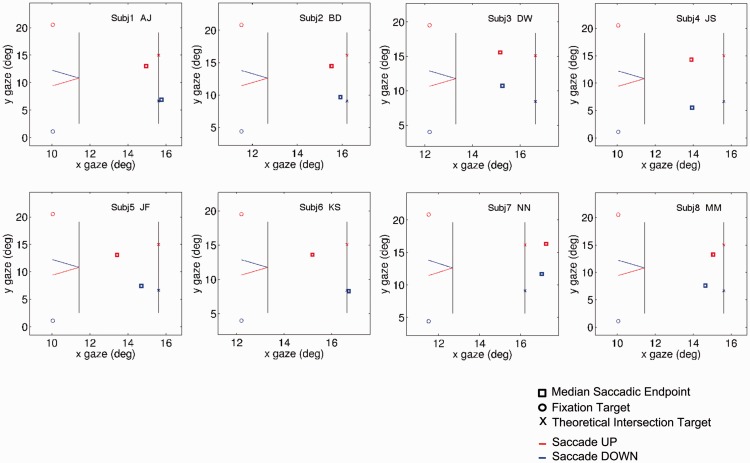
Experiment 3: The figure plots the median endpoints of saccades as rectangles (red, upwards pointing configuration; blue, downwards pointing). Initial fixation position is shown as an open circle, and target position as a cross. Each panel represents a single subject.

**Figure 7.**
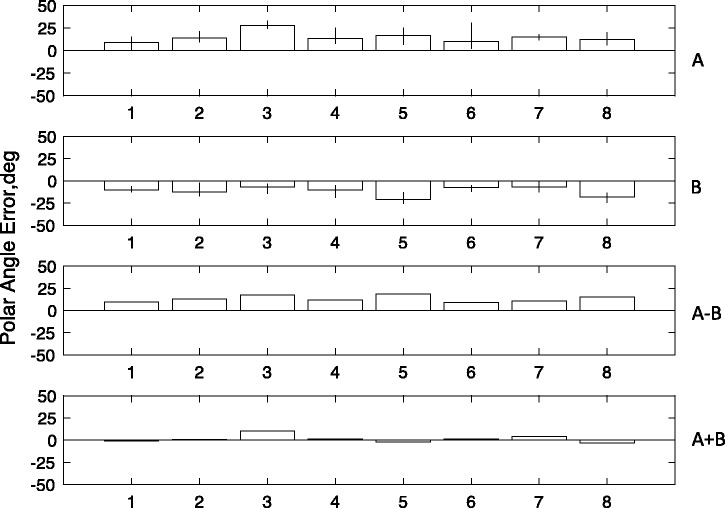
The figure shows the absolute angular error of the saccade with respect to the target angle from fixation (i.e., abs(target) − abs(saccade)). The top row (A) shows results from Experiment 3 where the FP3 was outside the figure. The middle row (B) shows results from Experiment 2 where the FP was at the tip of the pointer. The bottom row shows the difference between the two experiments, a measure of the putative P-bias. The bottom row shows the sum of the two experiments, a measure of the tendency to take the shortest path to the landing line.

Figure 6 shows the mean saccade endpoints. It is clear that there was substantial Y error in most subjects, along with X error in most participants as in the previous experiment. (For statistical analysis, see Appendix Figure A4). Figure 7 shows the polar angle of saccades, compared with the values in the previous experiment (Experiment 2). These are now in opposite directions between the two experiments, as would be predicted from a P-bias (A − B)/2, (13.06°, *t* = 13.06, *p* < .01), but there is no significant general bias (A + B)/2, (2.06°, *t* = 1.34, *p* > .1). The absolute magnitude of the P-bias in the two experiments is not significantly different, *t*(7) = 0.98; *p* > .1.

These results confirm that the eye movement bias in extrapolation is not entirely due to a tendency to take a shorter path across the gap. In addition, there is a tendency to take the path that would be predicted from the perceptual P-bias, that is, a path that takes the saccade to a target position that is not the point of intersection between the pointer and the landing line. The direction of the effect, like the perceptual P-bias, can be summarized by saying that the apparent path across the gap is shifted toward the horizontal, that is, in a direction orthogonal to the parallels. In thinking about this, it is important to distinguish between the path of the saccade and the imaginary path of the pointer across the gap. The path of the saccade is not necessarily shifted toward the right angle; indeed, as shown in the present experiment, it can be longer than the correct path.

It could be argued that the FP3 position not only alters the position of the target relative to fixation but also that of the pointer displacing the latter into a parafoveal position. If we had found no P-bias in this condition, eccentricity would have been a plausible explanation. However, Wenderoth, White, and Beh (1978) have shown that there is little effect of fixation positions on the perceptual Poggendorff effect, and this agrees with our finding that there is still a P-bias in the FP3 condition.

## Experiments 1 to 3 Summary

**Figure 8.**
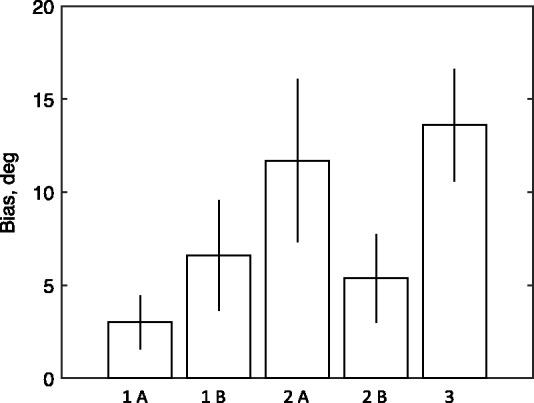
The vertical axis shows the mean bias over observers, and the 95% confidence intervals (vertical bars). The bias is the deviation of the saccade angle from the veridical angle, in the direction expected from the classical perceptual Poggendorff bias. The labels 1A, 1B, 2A, 2B, and 3 refer, respectively, to the collinear condition in Experiment 1, the orthogonal condition in Experiment 1, the condition with two parallels in Experiment 2, the condition with a single vertical line in Experiment 2, and to Experiment 3.

The key results of the experiments are summarized in Figure 8, which shows the means over participants in the various conditions, and their 95% confidence intervals. The bias in Figure 8 is defined as the bias expected if the saccade follows a *perceptual* path between the parallels that is a shorter path than the correct path to the landing line. This is in the same direction as the P-bias but should not be confused with it because it is only one possible cause of the P-bias. The full P-bias may include other causes that operate either in the same direction as the orthogonal construction bias (angular repulsion of the pointer) or in the opposite direction (e.g., Experiment 1A), where attraction of the saccade into the acute angle would lengthen the path to the landing line.

## General Discussion

The results of Experiment 1 confirm with saccadic eye movements that there is a mislocation of the pointer-vertical intersections in the Poggendorff figure, in the direction expected from the P-bias, and in agreement with psychophysical findings using the method of adjustment reported by Morgan (1999) and Morgan and Dillenburger (2016). The magnitude of this effect is considerably smaller than the P-bias found by extrapolation (Experiment 2) but it may contribute to it. The mislocation bias is consistent with neural blurring by second-order filters. One line of evidence supporting blurring is that increased optical blurring or low-pass filtering enhances the magnitude of the P-bias in the Poggendorff figure, as well as in its acute-angle amputated versions (Morgan, 1999).

We considered the possibility that biases are due to saccadic undershoot. The tendency for saccades to undershoot a target has been documented repeatedly for simple tasks such as saccading toward a single target dot (Becker & Fuchs, 1969), the center of a random dot pattern (McGowan, Kowler, Sharma, & Chubb, 1998), or to targets of different sizes (Ploner, Ostendorf, & Dick, 2004). These studies have in common that they indicated a center of mass effect on eye movement landing position. That is, saccades undershoot, but do so relative to the average center of a targeted area, as defined, for example, by visual boundaries (Findlay, Brogan, & Wenban-Smith, 1993), or possibly temporal set (Kapoula & Robinson, 1986). Comparable results were found in reading: Vitu (1991) reported that saccades tended to land short of a center of gravity of words, comparable to McGowan et al.’s study with random dot pattern. It has been argued, that undershooting may be a deliberate feature of the saccadic system, possibly to minimize flight time (Harris, 1995) and thereby to conserve energy, while aiming to land at a position sufficiently close (and thereby most informative) to the target. However, another possibility to undershoot as an explanation of the Poggendorff effect is that there is a bias toward taking a path that is orthogonal to the landing line. These two possibilities (undershoot vs. an orthogonal path) can be dissociated by varying the start point of the saccade, so that an orthogonal path becomes an overshoot (Experiment 3).

However, the large P-bias in extrapolation is not, as suggested earlier (Melmoth et al., 2015) a bias toward the saccade taking the shortest route to the landing line. When the FP is on the tip of the pointer, the P-bias and an undershoot cannot be distinguished, but when the FP is positioned (Experiment 3) so that a P-bias would make the saccade path longer (an overshoot) the P-bias is still found. For the same reason, the P-bias in saccades cannot be attributed to a tendency to cross the gap between the parallels at right angles. Instead, the P-bias appears to arise from a mislocation of the extrapolated target, similar to the perceptual P-effect.

A further finding of these experiments is that there is still a large P-bias when the left-hand vertical inducing line is absent. This agrees with the findings of Melmoth et al. (2015) and rules out the idea that expansion of the acute angle (Blakemore et al., 1970) is a complete explanation of the saccadic P-bias. There was a small but significant effect of the inducer on polar angle in Experiment 2 (confirming Melmoth et al., 2015) Therefore, the saccadic P-bias differs from the perceptual P-bias in the effect of the inducer, which is large for the perceptual effect (Hotopf & Hibberd, 1989; Melmoth et al., 2015). Melmoth et al. (2015) explained this larger P-bias for saccades with the saccadic undershoot, but we can now see that this is not correct. In Experiment 3, the P-bias is in the direction of an overshoot. The effect on saccades should be smaller, if it was largely due to the saccade’s inherent tendency to undershoot. However, a large P-bias is still found.

### Causes of Failure of Saccades to Reach the Landing line

In some cases in Experiment 2, this X error was dramatic. Subject JS in particular hardly moved his eyes from the FP at all, although he thought he had. This was not a general failure, because in Experiments 1 and 3, the same subject managed to move through at least 50% of the distance to the landing line.

### Relationship Between Psychophysical and Eye Movement Biases

The psychophysical results reported by Morgan and Dillenburger (2016) showed multiple causes of the P-bias. There were relatively minor contributions from (a) a mislocation bias in the position of oblique T-intersections; (b) an orientation bias in the pointer, local to an oblique T intersection; and (c) a bias in the orientation of virtual lines crossing the gap between two parallels. The latter could be considered either as a bias in the perceived virtual line, or as a construction bias in extrapolation. The present results with extrapolation eye movements show similar effects, as follows:

1. A mislocation bias. Experiment 1 shows a small tendency for saccades aimed at an oblique T to be directed toward the interior of the acute angle.
2. Eye movements directed to the extrapolated point of intersection between a pointer and landing line show a strong P-bias, consistent with orientation repulsion of the pointer (Experiment 2). However, unlike the psychophysical P-bias, this effect is only weakly dependent on the presence of the left-hand parallel and thus cannot be entirely due to orientation repulsion. There is thus a bias in the saccades to behave as if the pointer was orthogonal to the landing line, even when the oblique T-intersection is absent. The same phenomenon was reported by Melmoth et al. (2015) and mistakenly attributed to saccadic undershoot.
3. Experiments in which the starting position of the saccade was varied showed that the largest cause of the saccadic P-bias was not undershoot, but a bias for the extrapolation eye movement to follow the direction of the perceptual P-bias. In discussing the possible origins of this P-bias in the accompanying paper (Morgan & Dillenburger, 2016), we considered two possibilities. One is that the perceived angle of the virtual line across a gap between two parallels is biased toward the orientation of the parallels. The other is that there is a bias *in constructing* a virtual line across a gap, in a direction orthogonal to the orientation for the parallels. The eye movement findings argue in favor of the second of these alternatives, since there were no explicit target features on the landing line. In their absence, the observer was forced to carry out an extrapolation and the resulting bias is therefore one in extrapolation.

The explanation of the orthogonal extrapolation bias can at present be only a matter for speculation. It is in the same direction as the “orthogonal orientation bias” reported by Morgan, Medford, and Newsome (1995) but that effect was found with real lines and only with small gaps between the parallels. A Bayesian interpretation of the bias (Knill & Richards, 1996) would begin with the fact that the actual position of the extrapolated intersection is unknown to the observer and is subject to sensory noise. This is supported by the psychophysically measured values of the dispersion (s) of the psychometric function, which has a value in the region of 3°. (e.g., a mean value over nine observers of 2.7° in the control condition for aligning two pointers with a gap between). Assuming that the likelihood function for extrapolated position is not biased, the bias must arise from a prior distribution favoring the shorter paths across the gap. This would make sense for minimizing the effort of a motor response, but as we have seen, it can lead to eye movements taking a *longer* path to the landing line. However, this was in the specific and unusual case of a starting point for the saccade outside the figure. The more general case may be that the bias reduces the energy expended on motor behavior. A similar analysis can be made for the general undershoot behavior found for saccades. It is tempting to see the act of perceptual extrapolation as a form of internal motor behavior, which is subject to the same general rule of minimization of path.

Another explanation may be a tendency to make horizontal rather than oblique saccades. There is evidence from Becker and Juergens (1990), as well as from scene viewing and other tasks, that people tend to make more horizontal eye movements, and that oblique and vertical saccades are subject to more errors and are essentially “more difficult” to program. However, this explanation is countered by the results of Experiment 3, where subjects made a more oblique saccade as a result of the P-bias. The bias in this case was not statistically different from that in Experiment 2, where the P-bias led to a less oblique saccade.

Spering and Carrasco (2015) have recently reviewed dissociations between visual perception and eye movements, for example, greater sensitivity of smooth pursuit than perception to threshold changes in target velocity (Tavassoli & Ringach, 2010). Our data show a large degree of communality between the perceptual and the saccadic P-bias but also some differences. The most important is that the influence of the left-hand inducing line, and its oblique T-intersection with the parallel, is larger in the case of the perceptual P-bias.

## Supplementary Material

Supplementary material

## Figures and Tables

**Figure A5.**
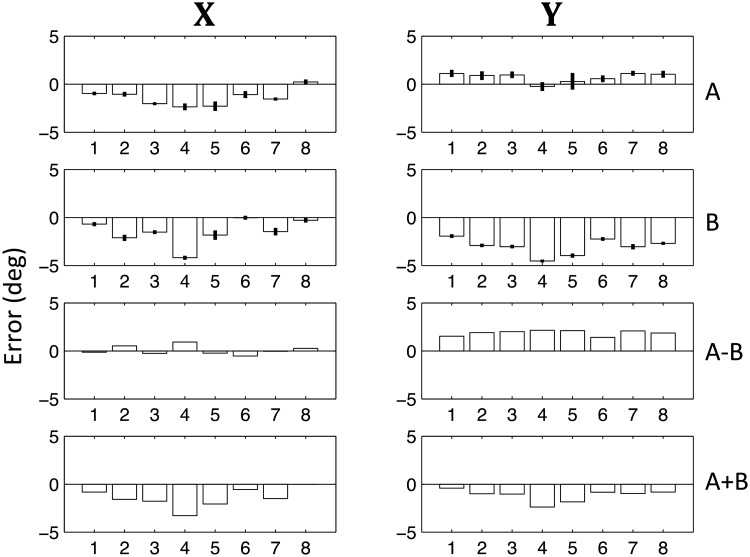
Results of Experiment 3. The figure shows the median and interquartile range of saccadic overshoots, separately for the X (left) and Y (right) dimensions in space. The means for the upward- and downward-pointing configurations are combined into a single grand mean. Within each panel, the height of each bar represents the extent of overshoot from the nominal target in deg VA for each of the six subjects who completed all conditions. The error bars show interquartile range. The first row (a) shows the results for Experiment 3 where the FP was outside the figure. The second (b) row shows results for Experiment 2 where the FP was at the tip of the pointer. The third row shows the mean difference between the two experiments, a measure of the putative P-bias. The fourth row shows the sum of the two experiments, a measure of undershoot. The undershoots (A + B) were significant in X (−1.45° VA, *p* < .01) and in Y (−1.16° VA, *p* < .01). The P-bias (A − B) was not significant in X (0.06° VA) but was highly significant in Y (1.88° VA, *p* < .01).

## Author Note

Author Barbara Dillenburger is now affiliated with Division of Optometry and Visual Science, Institute of Health Sciences, City University London, London, UK.

## Supplemental Material

The online supplements are available at http://journals.sagepub.com/doi/suppl/10.1177/2041669517699221.

## Appendix

### Drift- and Offset-Correction

Even the fixations recorded very soon after calibration can differ substantially from the calibrated center of the screen, as may be shown in Figure A1, where the black traces show the recorded fixation positions on each trial expressed as deviations from the calibrated screen center. In a comparison of systematic errors in eye tracking across 16 subjects, Hornof and Halverson (2002) found systematic offsets across different target locations within the range of +/−2° of visual angle. In a comparable condition (Experiment 2, saccade from a fixation spot to a single target point), our eight subjects showed comparable offsets during initial fixation from calibration of on average 1.44° +/− 0.83 (X) and −0.15° +/− 0.58 (Y). Offsets varied with experimental condition and subject but were in general in the range of previous reports. This initial deviation can be followed by slow drifts. The reasons for the large initial discrepancies from calibration are not clear but may include changes in pupil size, for example, due to state of arousal, which have a large effect on recorded gaze position (Gabay, Pertzov, & Henik, 2011; Wildenmann & Schaeffel, 2013; Wyatt, 2010). Different methods for online and post hoc correction are in debate (Choe, Blake, & Lee, 2016; Hornof & Halverson, 2002; Wildenmann & Schaeffel, 2013) but depend on the specific experiment design. We employed a post hoc method to compensate for systematic errors and slow drifts. Given that we have only one central fixation target, in addition to saccade targets, which are possibly affected by the Poggendorff effect (and can thus not be used as a known target location), we are limited to correcting gaze space by centering it on the same initial fixation point. Therefore, the raw gaze positions of fixations to the central fixation spot over the entire experiment (black traces in Figure A1(a) and (b)) were, independently for X and Y, smoothed by means of a sliding average with a window size of five trials (red curve in Figure A1(a) and (b)). Outliers (seen as large spikes in Figure A1(a) and (b)) were truncated to a value of 1 standard deviation of the mean (*SEM*) measured over the entire recording block (see dashed horizontal lines in Figure A1(a) and (b) for +/− 1 *SEM*). The moving average was then subtracted from the gaze positions on each trial to yield a corrected value centered on the location of the fixation target (blue line in Figure A1(a) and (b)). The same correction was applied to every point on the subsequent saccade (blue open circles in Figure A1(c) and (d)).

While we did not specifically correct for pupil size variations and their effect on apparent gaze position (Choe et al., 2016, Wildenmann & Schaeffel, 2013; Wyatt, 2010), our drift correction does partially correct for the pupil size artifact. Our correction method uses deviations of gaze from central fixation at the end of the initial fixation period, that is, in the range of 50 to 200 ms before saccade onset. With typical latencies of pupil size changes in the range of hundreds of ms (Bergamin, 2003), any deviations from calibrated gaze position due to pupil size differences are not likely to change during the trials’ saccade and can thus be corrected for by our algorithm.

### Exclusion Procedure

Trials were also excluded if any of the following parameters deviated by more than two standard deviations from the mean:

- Saccade amplitude (distance between start and end point)
- Summed total distance (sum of distances between each sample to the next over the entire saccade)
- Start position
- Velocity peak time point
- Summed total velocity (sum of velocities between each sample to the next over the entire saccade)

These exclusion parameters were chosen to detect saccades that had started at a fixation spot closer to the target, that is, were both too short and too far off the central fixation (as shown by the red trajectories in Figure A2(a)). Such short saccades, as well as turnaround saccades—as can be seen with the blue trajectory in Figure A2(a), and corresponding blue velocity profile in Figure A2(d)—could furthermore deviate in velocity peak time from the average saccade. In addition, measurement errors would rarely result in saccades that had a larger summed velocity or distance, as can be observed in the green trajectories in Figure A2(a), that show several deviations from the typically *smooth* movement pattern of a saccade.
