# Supplementary material for: Saccades to Explicit and Virtual Features in the Poggendorff Figure Show Perceptual Biases
Source: Iperception. 2017 Apr 21;8(2):2041669517699221. doi: 10.1177/2041669517699221 (PMC5407530; doi:10.1177/2041669517699221)
Supplement: Supplementary material [file IPE699221_supplementary_material.pdf]

Supplementary Material

<u>Drift- and Offset-correction</u>

Even the fixations recorded very soon after calibration can differ substantially from the calibrated center of the screen, as may be seen from Fig. S1, where the black traces show the recorded fixation positions on each trial expressed as deviations from the calibrated screen center. In a comparison of systematic errors in eye tracking across 16 subjects, Hornof & Halverson (2002) found systematic offsets across different target locations within the range of +- 2º of visual angle. In a comparable condition (Experiment 2, saccade from a fixation spot to a single target point) our 8 subjects showed comparable offsets during initial fixation from calibration of on average 1.44º +- 0.83 (X) and -0.15º +- 0.58 (Y). Offsets varied with experimental condition and subject, but were in general in the range of previous reports. This initial deviation can be followed by slow drifts. The reasons for the large initial discrepancies from calibration are not clear, but may include changes in pupil size e.g. due to state of arousal, which have a large effect on recorded gaze position (Gabay, Pertzov, & Henik, 2011; Wildenmann & Schaeffel, 2013; Wyatt, 2010). Different methods for online and post-hoc correction are in debate (Hornof & Halverson, 2002, Wildenmann & Schaeffel, 2013, Choe et al. 2016), but depend on the specific experiment design. We employed a post-hoc method to compensate for systematic errors and slow drifts. Given that we have only one central fixation target, in addition to saccade targets, which are possibly affected by the Poggendorff effect (and can thus not be used as a known target location), we are limited to correcting gaze space by centering it on the same initial fixation point. Therefore, the raw gaze positions of fixations to the central fixation spot over the entire experiment (black traces in Fig. S1 A&B) were, independently for X and Y, smoothed by means of a sliding average with a window size of 5 trials (red curve in Fig. S1 A & B). Outliers (seen as large spikes in Fig. S1 A & B) were truncated to a value of 1 standard deviation of the mean (SEM) measured over the entire recording block (see dashed horizontal lines in Fig. S1 A & B for +/- 1 SEM). The moving average was then subtracted from the gaze positions on each trial to yield a corrected value centered on the location of the fixation target (blue line in Fig. S1 A & B). The same correction was applied to every point on the subsequent saccade (blue open circles in Fig. S1 C & D).

Fig. S1 about here

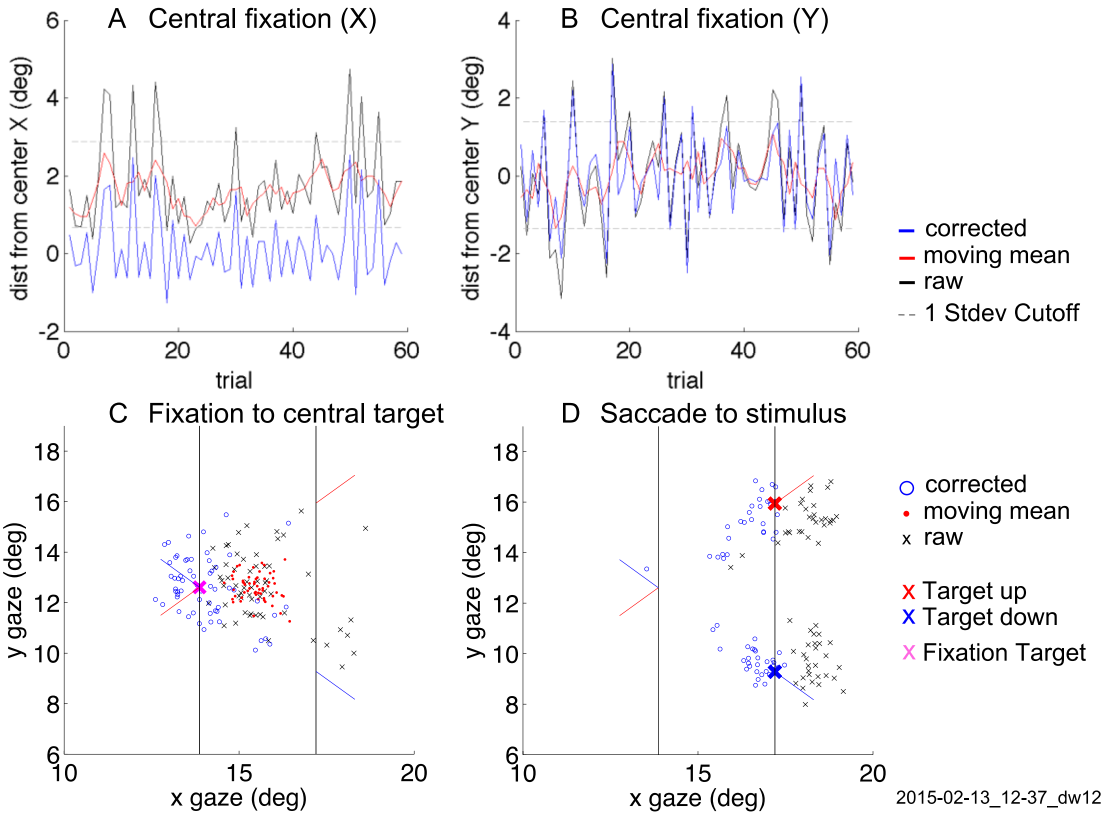

Fig. S1. Example of drift and head movement correction of a single test run with 60 trials. (A) Horizontal distance (gaze coordinates, deg of visual angle) of subject's fixation across trials from the calibrated central fixation target; B) Vertical distance (deg) of subject's fixation across trials from the calibrated central fixation target; black traces in (A) and (B) show the raw eye movement data, red traces show the moving mean (smoothing window of 5 trials, cropped to 1 standard deviation of the mean, as indicated by the horizontal dashed lines) over all 60 trials. The blue trace shows the corrected gaze positions. In (C) and (D), initial fixation (C) and endpoint of the first saccade (D) are shown superimposed on the stimulus configuration (Expt 1) , with gaze coordinates plotted in deg. Raw eye positions are shown as black crosses, moving mean thereof in red (C), and corrected eye positions as blue open circles (C &D). Initial fixation location is indicated by the pink cross (C), saccade targets as red (upward condition) and blue (downward condition) crosses (D).

While we did not specifically correct for pupil size variations and their effect on apparent gaze position (Choe, Blake, & Lee, 2016, Wyatt, 2010, Wildenmann & Schaeffel, 2013), our drift correction does partially correct for the pupil size artifact. Our correction method uses deviations of gaze from central fixation at the end of the initial fixation period, i.e. in the range of 50-200 msec before saccade onset. With

typical latencies of pupil size changes in the range of hundreds of msec (Bergamin, 2003), any deviations from calibrated gaze position due to pupil size differences are not likely to change during the trials' saccade and can thus be corrected for by our algorithm.

Exclusion Procedure

Trials were also excluded if any of the following parameters deviated by more than 2 standard deviations from the mean:

- Saccade amplitude (distance between start and end point)
- Summed total distance (sum of distances between each sample to the next over the entire saccade)
- Start position
- Velocity peak time point
- Summed total velocity (sum of velocities between each sample to the next over the entire saccade)

These exclusion parameters were chosen to detect saccades that had started at a fixation spot closer to the target, i.e. were both too short and too far off the central fixation (as shown by the red trajectories in Fig. S2A). Such short saccades, as well as turnaround saccades - as can be seen with the blue trajectory in Fig. S2A, and corresponding blue velocity profile in Fig. S2D - could furthermore deviate in velocity peak time from the average saccade. In addition, measurement errors would rarely result in saccades that had a larger summed velocity or distance, as can be observed in the green trajectories in Fig. S2A, that show several deviations from the typically 'smooth' movement pattern of a saccade.

Fig. S2 about here

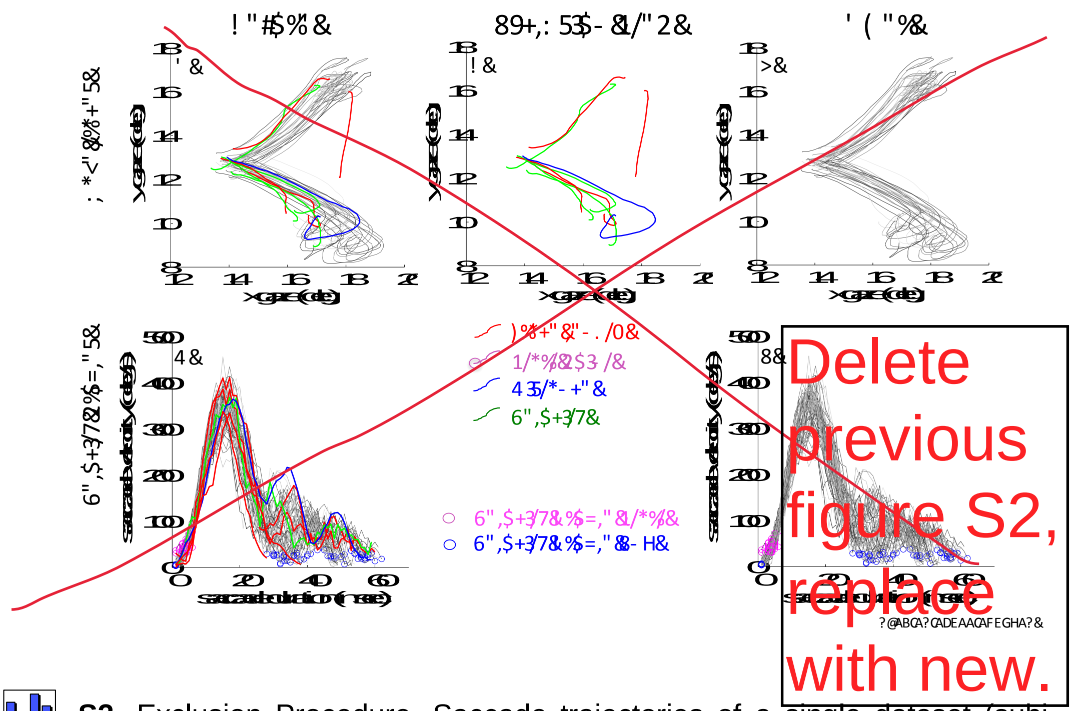

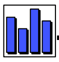. **S2.** Exclusion Procedure. Saccade trajectories of a single dataset (subj. BD, experiment 1, 60 trials) are plotted in gray (A) before and (C) after the exclusion step in the top row. Velocity profiles are shown in gray, (D) before and (E) after exclusion, respectively, in the bottom row. In the middle graph, (B) trajectories of excluded saccade are plotted colored according to the parameter in which they deviated by more than 2 stdev from the average saccade: red: trace length; pink: starting point; blue: summed distance; green: peak velocity/summed velocity. Note that traces are color-coded according to the first deviating parameter, if they differed from the average in several aspects. In (D&E), velocity profile based saccade starts are marked as pink, saccade ends as blue open circles.

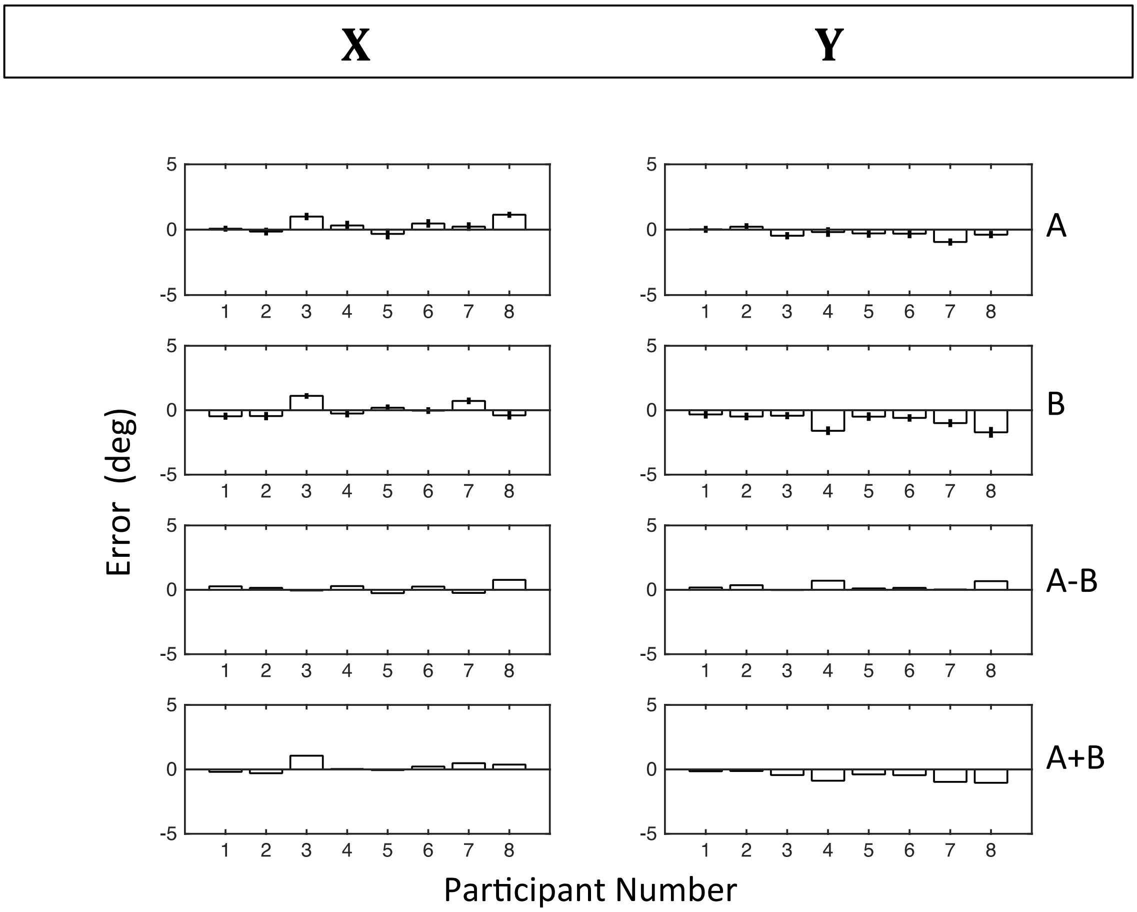

Fig. S3. Results of Experiment 1. The figure shows the median and interquartile range of saccadic errors, separately for the X (left) and Y (right) dimensions in space. The results for upright and inverted configurations are combined into a single grand median. Within each panel the height of each bar represents the error from the nominal target in deg VA for each of the 8 subjects who completed all conditions. By convention, negative values mean that the value is less than it would have been had the saccadelanded on target. The error bars show inter-quartile range. The first row shows (A) the results for the collinear configuration (see Fig. 4). The second row (B) shows results for the orthogonal configuration. The third row shows the difference Collinear-Orthogonal, a measure of the acute-angle bias without the influence of undershoot ($t=2.5$, $p< 0.05$). The fourth row shows the sum Collinear+Orthogonal, a measure of the undershoot ($t=3.6$, $p< 0.01$).

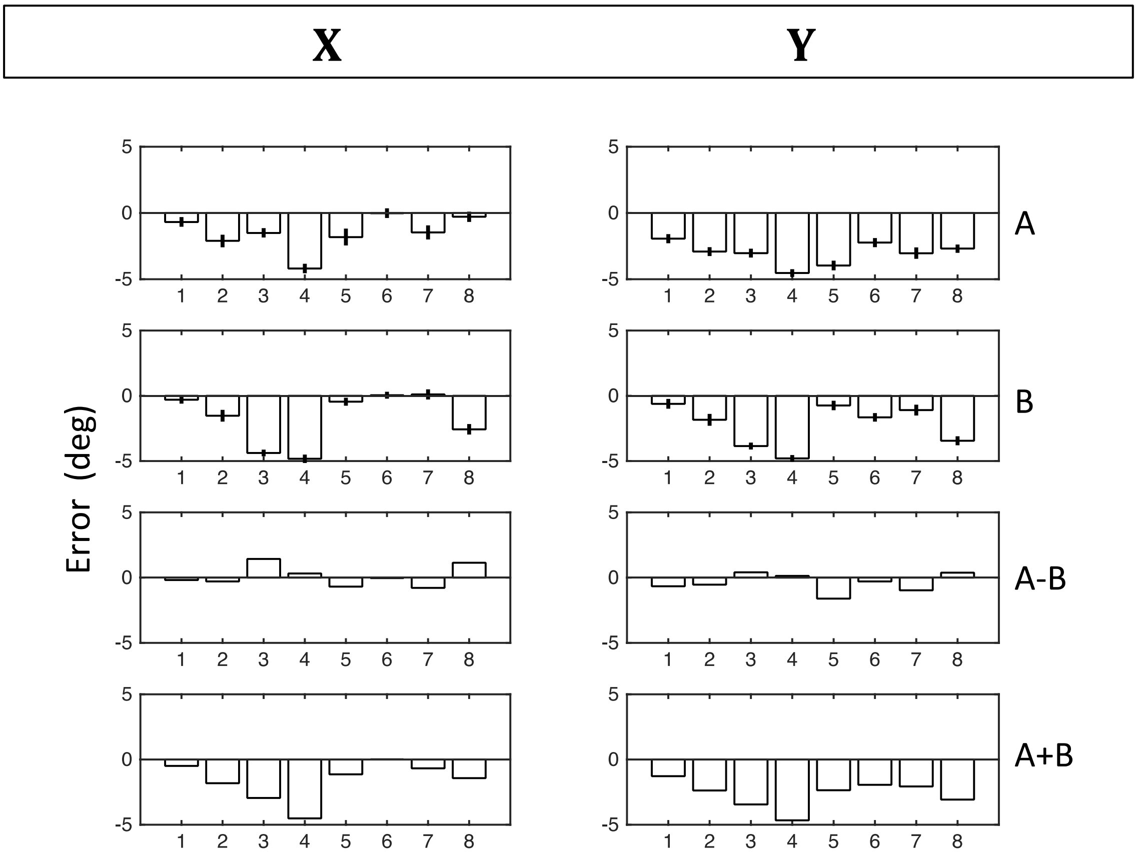

Fig. S4. Results of Experiment 2. The figure shows the median and interquartile range of saccadic overshoots, separately for the X and Y dimensions in space. The means for the upward- and downward-pointing configurations are combined into a single grand mean. Within each panel the height of each bar represents the extent of overshoot from the nominal target in deg VA for each of the 6 subjects who completed all conditions. The error bars show inter-quartile range. The first row shows the results for the condition where the left-hand vertical inducer was present. The second row shows results for the control condition where the left-hand vertical

was absent. The third row shows the mean difference between the two conditions, a measure of the putative P-bias due to the vertical. The fourth row shows the sum of the two conditions, a measure of undershoot. The Figure shows that there were undershoots in the expected direction both with the left-hand vertical inducer present and absent. However, these are largely accounted for by a general undershoot (A+B)/2 rather than by a P-bias (A-B)/2. The X shift due to the inducing line (A-B) was 0.114 deg VA (t=0.5, p=0.7), the Y shift was -0.39 deg VA (t=0.2, p> 0.5). The overall undershoot (A+B) was -1.6 (t=2.05, P< 0.5) deg VA for X and -2.65 (t=3.29, p< 0.001) deg VA for Y.

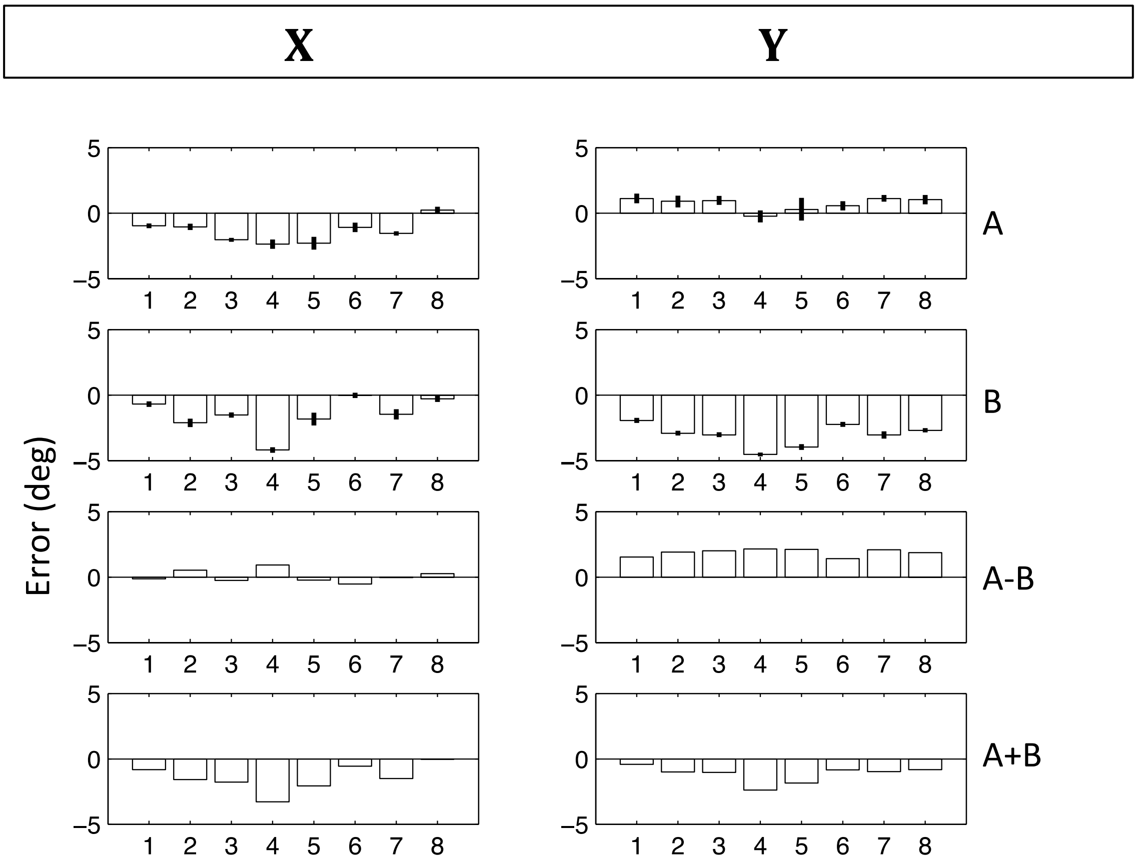

Fig. S5. Results of Experiment 3. The figure shows the median and interquartile range of saccadic overshoots, separately for the X (left) and Y (right) dimensions in space. The means for the upward- and downward-pointing configurations are combined into a single grand mean. Within each panel the height of each bar represents the extent of overshoot from the nominal target in deg VA for each of the 6 subjects who completed all conditions. The error bars show inter-quartile range. The first row (A) shows the results for Experiment 3 where the FP was outside the figure. The second (B) row shows results for Experiment 2 where the FP was at the tip of the pointer. The third row shows the mean difference between the two experiments, a measure of the putative P-bias. The fourth row shows the sum of the two experiments, a measure of undershoot. The undershoots (A+B) were significant in X (-1.45 deg VA, $p < 0.01$) and in Y (-1.16 deg VA, $p < 0.01$). The P-bias (A-B) was not significant in X (0.06 deg VA) but was highly significant in Y (1.88 deg VA, $p < 0.01$).
